# Single-cell profiling of peripheral immune remodeling in BK polyomavirus–associated nephropathy after kidney transplantation

**DOI:** 10.3389/fimmu.2026.1939071

**Published:** 2026-10-01

**Authors:** Hyunjoo Bae, Eun Jeong Ko, Byung Ha Chung, Eun-Jee Oh

**Affiliations:** 1 Department of Medical Sciences, Graduate School of The Catholic University of Korea, Seoul, Republic of Korea; 2 Department of Laboratory Medicine, Seoul St. Mary’s Hospital, College of Medicine, The Catholic University of Korea, Seoul, Republic of Korea; 3 Division of Nephrology, Department of Internal Medicine, The Catholic University of Korea, Bucheon St. Mary’s Hospital, Bucheon, Republic of Korea; 4 Division of Nephrology, Department of Internal Medicine, Seoul St. Mary’s Hospital, College of Medicine, The Catholic University of Korea, Seoul, Republic of Korea; 5 Research and Development Institute for In Vitro Diagnostic Medical Devices, The Catholic University of Korea, Seoul, Republic of Korea

**Keywords:** BK polyomavirus-associated nephropathy, kidney transplantation, peripheral immune remodeling, pseudobulk analysis, rare immune populations, single-cell RNA sequencing

## Abstract

BK polyomavirus–associated nephropathy (BKPyVAN) remains a major cause of allograft dysfunction after kidney transplantation. To define peripheral immune signatures associated with distinct clinical states of BKPyV infection, we performed single-cell RNA sequencing of peripheral blood mononuclear cells from kidney transplant recipients across three clinical states: stable graft function (n = 1), BKPyV DNAemia (n = 2), and biopsy-proven BKPyVAN (n = 3). We identified 17 immune cell populations and observed remodeling of both the innate and adaptive compartments. Monocyte subsets differed across clinical states, with CD16^+^ non-classical monocytes enriched in BKPyV DNAemia and inflammatory monocytes more abundant in BKPyVAN. CD8^+^ T-cell frequency was higher in BKPyVAN than in BKPyV DNAemia, while NK-cell frequency was lower in BKPyV DNAemia and BKPyVAN than in Stable, consistent with altered antiviral surveillance. A rare but transcriptionally distinct population, termed Activated Effector Cells (AECs), comprised only 0.60% of cells in BKPyVAN (104 of 17,238 cells) yet accounted for 12.15% of cluster-associated marker calls, the highest among the 17 populations, and displayed marked enrichment of cell cycle and RNA metabolic pathways. Across multiple lineages, AUC-based gene set enrichment analysis revealed convergent activation of RNA processing and intracellular transport pathways. Pseudobulk differential expression and permutation testing supported condition-associated transcriptional signatures, and leading-edge gene analysis identified candidate transcriptional readouts, including cell cycle regulators, inflammatory mediators, and RNA processing genes, with potential utility for blood-based monitoring. These findings reveal peripheral immune remodeling across the clinical states of BK polyomavirus infection and highlight the value of resolving rare, transcriptionally distinct immune populations, providing a rationale for noninvasive, transcriptomics-informed monitoring and risk stratification strategies.

## 1 Introduction

Kidney transplantation remains the most effective treatment for end-stage renal disease, but long-term graft survival is limited by complications such as BK polyomavirus–associated nephropathy (BKPyVAN), which occurs in up to 10% of recipients and represents a leading cause of late allograft loss (1–5). BKPyVAN results from reactivation of latent BK polyomavirus (BKPyV) under immunosuppression, leading to viral replication, tubular epithelial injury, and progressive nephropathy. Current management is largely restricted to empiric reduction of immunosuppression, which often increases the risk of rejection (6, 7), underscoring the need for precise, mechanism-based monitoring and intervention strategies.

Despite its clinical importance, the immunopathogenesis of BKPyVAN remains incompletely understood. Although CD8^+^ cytotoxic T cells, NK cells, and monocytes have been implicated in antiviral defense, their specific roles, activation states, and interplay in the peripheral compartment across BKPyV clinical states remain poorly defined (8–12). In particular, most prior studies have relied on bulk transcriptomic or flow cytometric analyses, which lack the resolution to capture rare or transient immune subsets that may drive disease outcomes and may obscure cell state-specific signals through population averaging (13–15). More recent single-cell studies have begun to characterize BKPyV-associated cellular heterogeneity in renal epithelial systems and infected kidney tissue, including differences in viral transcriptional burden, cell-cycle programs, stress responses, and epithelial–immune interactions (16, 17). However, these studies have focused primarily on renal epithelial or intrarenal compartments, and peripheral immune remodeling across BKPyV DNAemia and biopsy-proven BKPyVAN remains poorly characterized.

Single-cell RNA sequencing (scRNA-seq) resolves immune heterogeneity at high resolution, enabling detection of previously unrecognized rare or transitional immune states and their gene expression programs (18–22). In kidney transplantation, scRNA-seq has been used to dissect alloreactive or rejection-associated immune landscapes (18), yet few studies have applied this approach to virus-induced complications like BKPyVAN, especially using peripheral blood as a noninvasive immunologic window.

In this exploratory study, we applied scRNA-seq to peripheral blood mononuclear cells (PBMCs) from kidney transplant recipients spanning three clinical states: stable graft function, BKPyV DNAemia, and biopsy-proven BKPyVAN. Our goal was to define immune cell states and transcriptional programs associated with each clinical state. We specifically aimed to identify rare or transcriptionally distinct immune populations and to delineate cell type-specific pathway programs using an AUC-based gene set enrichment analysis (GSEA) approach, enabling extraction of leading-edge genes as exploratory transcriptional readouts.

Our study observed a rare Activated Effector Cell (AEC) population with a marked proliferation-associated transcriptional program, alongside clinical-state-associated changes in monocyte subsets and cytotoxic T cells. These findings suggest altered antiviral immune surveillance in individuals with BKPyVAN and provide a basis for monitoring and risk stratification strategies.

## 2 Materials and methods

### 2.1 Study population

Six kidney-transplant recipients were selected from patients enrolled at Seoul St. Mary’s Hospital Transplant Center between October 2018 and January 2021, based on the availability of peripheral blood mononuclear cell (PBMC) samples suitable for single-cell RNA sequencing. PBMCs were collected 1–8 months post-transplantation. Patients were classified into three clinical groups: stable graft function (Stable, n = 1), BKPyV DNAemia (n = 2, defined as two or more consecutive plasma BKPyV DNA measurements >1,000 copies/mL without biopsy-proven nephropathy; allograft biopsy was not clinically indicated, and these patients were not diagnosed with BKPyVAN in subsequent clinical records), and biopsy-proven BKPyVAN (n = 3). This study was approved by the Institutional Review Board of Seoul St. Mary’s Hospital (IRB #KC19SESI0061), and written informed consent was obtained from all participants in accordance with the Declaration of Helsinki.

### 2.2 PBMC preparation and single-cell RNA sequencing

Cryopreserved PBMCs were thawed, washed, and processed according to standard protocols. Cells were labeled with Cell Multiplexing Oligos (CMOs) per the 10x Genomics protocol. Three samples were pooled per multiplexing batch across two multiplexing batches. Single-cell RNA libraries were prepared using the Chromium Single Cell 3′ v3.1 platform (10x Genomics) and sequenced on an Illumina HiSeq X Ten system (150 bp paired-end; ~30,000 read pairs per cell). Detailed protocols are provided in the **Supplementary Materials and Methods**.

### 2.3 Data processing and quality control

Raw sequencing data were processed using Cell Ranger (v6.0.2; 10x Genomics) with the GRCh38 reference transcriptome (10x Genomics, version 2020-A). Samples were demultiplexed via the *cellranger multi* pipeline. In total, 31,779 high-quality cells were obtained across all samples. Detailed sequencing metrics are provided in **Supplementary Table S1** and **Supplementary Figure S1**.

### 2.4 Single-cell RNA sequencing data analysis

Downstream analyses were performed using Seurat (v5.1.0). Data were normalized using SCTransform, and datasets from different samples and sequencing runs were integrated using reciprocal PCA-based integration to mitigate batch effects (**Supplementary Figure S2**). Clustering was performed using a shared nearest neighbor (SNN) graph-based method with UMAP for visualization. Cell type annotation was performed based on canonical marker gene expression and established PBMC reference datasets, including the Human Cell Atlas. As a descriptive metric, the cluster-associated marker contribution was calculated for each cluster within each clinical condition as the number of Bonferroni-significant cluster-associated marker calls divided by the total number of such calls across all clusters within that condition, expressed as a percentage. Detailed methods are described in the **Supplementary Materials and Methods**.

### 2.5 Differential expression and pathway analysis

Cell type-specific differential expression across disease states was performed using the MAST model implemented in Seurat. We performed pathway-level analysis using an AUC-based GSEA approach (fgsea v1.22.0) (23) with gene sets from MSigDB (Hallmark, Reactome, KEGG, GO: BP; size 15–500). Detailed methodological considerations, including NES distribution characteristics, are described in the **Supplementary Materials and Methods** (**Supplementary Figure S3**). Pseudobulk differential expression analysis was performed by aggregating UMI counts per patient and cluster using DESeq2 (v1.42.0; design: ∼ cluster + condition) (24). The comparison between BKPyVAN and BKPyV DNAemia identified 1,825 significant DEGs (FDR < 0.05; **Supplementary Table S2**). A label-permutation test (n = 100) yielded an empirical p-value of 0.0099 (**Supplementary Table S3**). Given the limited sample size, analyses were designed to emphasize consistency across cell populations. Because AECs were rare outside BKPyVAN, AEC pathway enrichment was assessed within BKPyVAN samples using AUC-ranked GSEA rather than by comparing AECs across clinical conditions. As a lineage-restricted sensitivity analysis, AECs were additionally compared with CD8+ cytotoxic T cells within BKPyVAN using the same AUC-ranked GSEA framework.

## 3 Results

### 3.1 Patient characteristics and clinical stratification

Six kidney transplant recipients were enrolled, representing three distinct clinical phenotypes: stable allograft function (Stable, n = 1), asymptomatic BKPyV DNAemia (BKPyV DNAemia, n = 2), and BKPyVAN (n = 3). Both patients with BKPyV DNAemia developed detectable BKPyV replication early post-transplantation (day 32 and 63) while maintaining stable renal function. In contrast, BKPyVAN patients exhibited viral reactivation at variable timepoints (days 25, 92, and 132) followed by tissue-invasive disease confirmed by histopathology and positive SV40 staining. According to Banff criteria, two patients (BKPyVAN_1 and BKPyVAN_3) showed class 2 nephropathy and one (BKPyVAN_2) displayed class 1 nephropathy. Among patients with BKPyVAN, plasma BKPyV DNA levels at the time of PBMC sampling ranged from 7.97 × 10³ to 2.3 × 10^7^ copies/mL, with variable histopathologic severity. Clinical characteristics are summarized in **Table 1**.

**Table 1:** Clinical characteristics of kidney transplant recipients.

Characteristic	Stable	BKPyV DNAemia_1	BKPyV DNAemia_2	BKPyVAN_1	BKPyVAN_2	BKPyVAN_3
Sex	M	M	F	M	F	F
Age (years)	61	40	28	59	65	30
ABO compatibility	ABOc	ABOc	ABOc	ABOi	ABOc	ABOc
HLA mismatches (n)	3	3	3	4	3	2
Induction therapy: basiliximab	+	+	+	−	+	+
Anti-thymocyte globulin (ATG)	−	−	−	+	−	−
Baseline comorbidities	DM	HTN	DM	Unknown	DM	Chronic GN
Panel-reactive antibody (PRA), % (class)	16.6 (class I)	NA	40.0 (class II)	NA	8.3 (class I)	17.0 (class I)
Onset of BKPyV DNAemia (days post-transplant)	NA	32	63	92	132	25
Plasma BKPyV DNA load at sampling (copies/mL)	NA	866	7,680	7,970	2.3 × 10^7^	7.08 × 10^6^
BKPyV nephropathy (Banff class)	−	−	−	Class 2	Class 1	Class 2
PBMC sampling time (days post-transplant)	28	46	103	215	230	111
Time from BKPyV DNAemia onset to PBMC sampling (days)	NA	14	40	123	98	86
Immunosuppressive regimen at PBMC sampling	TAC + MPA + DFZ	CsA + PSL	TAC + MPA + DFZ	CsA + DFZ	CsA + DFZ + LEF	CsA + DFZ + LEF

BKPyV, BK polyomavirus; BKPyVAN, BK polyomavirus-associated nephropathy; ABOc, ABO-compatible; ABOi, ABO-incompatible; ATG, anti-thymocyte globulin; PRA, panel-reactive antibody; PBMC, peripheral blood mononuclear cell; DM, diabetes mellitus; HTN, hypertension; GN, glomerulonephritis; NA, not applicable; TAC, tacrolimus; CsA, cyclosporine; MPA, mycophenolic acid; DFZ, deflazacort; PSL, prednisolone; LEF, leflunomide.

### 3.2 Single-cell transcriptomic landscape across disease states

Single-cell RNA sequencing of PBMCs yielded 31,779 cells across the three groups: Stable (5,473 cells), BKPyV DNAemia (9,068 cells), and BKPyVAN (17,238 cells). Unsupervised clustering and UMAP visualization identified 17 transcriptionally distinct immune populations (**Figure 1A**), including CD8^+^ cytotoxic T cells, CD4^+^ T cell subpopulations, NK cells, and a spectrum of myeloid cells such as classical monocytes (CD14^+^), non-classical monocytes (CD16^+^), inflammatory monocytes and macrophages. Canonical marker expression supported biologically coherent identities, with CD8A and GZMH in cytotoxic T cells, MS4A1 and CD79A in B cells, and FCGR3A and APOE in non-classical monocytes (**Figure 1B**). Cell distributions by clinical state are shown in **Supplementary Figure S4**.

**Figure 1:**
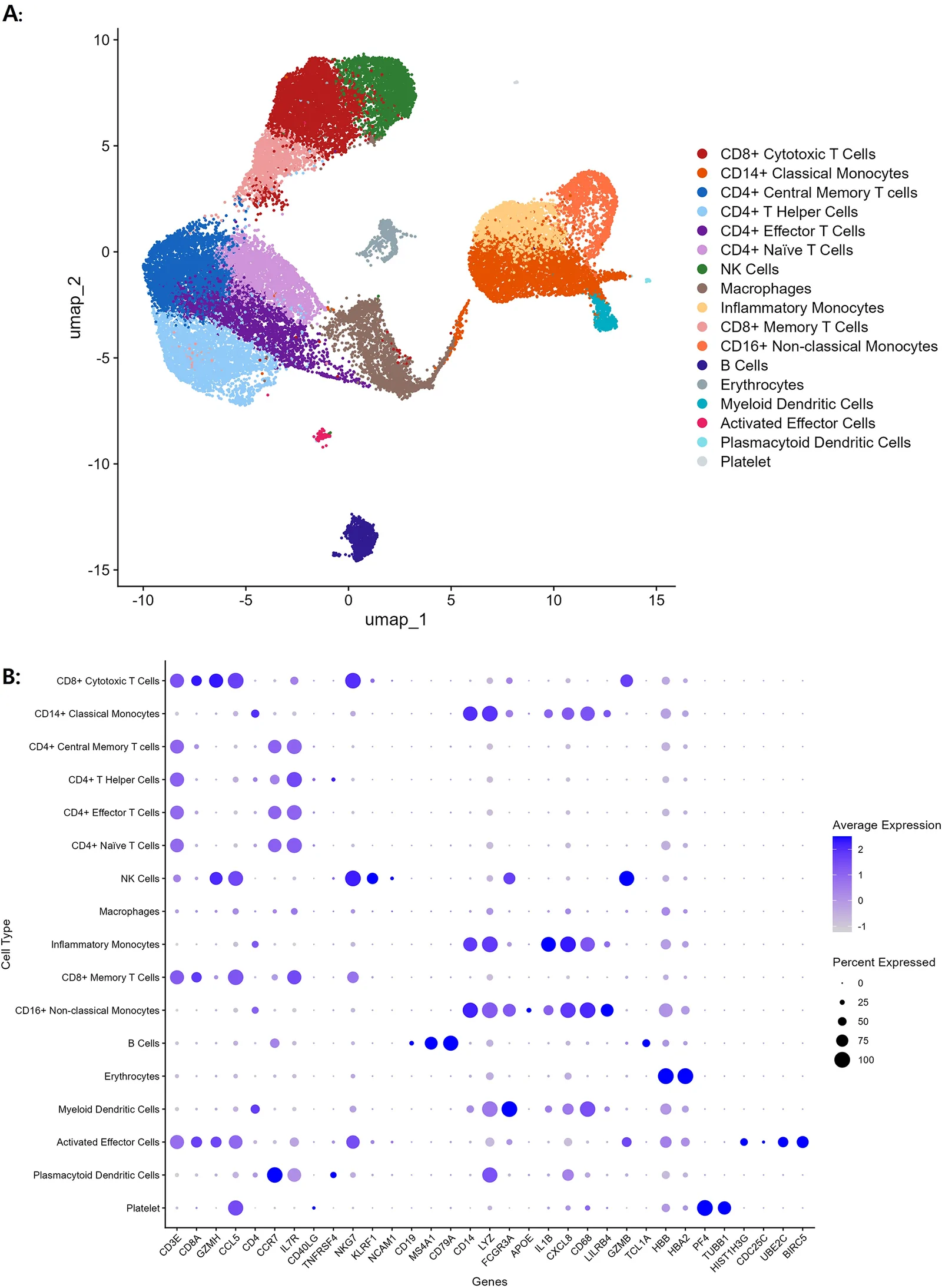
Single-cell immune profiling of peripheral blood from Stable, BKPyV DNAemia, and BKPyVAN patients. **(A)** UMAP projection of 31,779 single PBMCs across all samples, colored by immune cell identity. Seventeen transcriptionally distinct immune populations were identified based on canonical marker expression and unsupervised clustering. **(B)** Dot plot illustrating scaled average expression and percentage of cells expressing canonical marker genes across annotated immune cell types. Dot size reflects the proportion of cells expressing each gene; color intensity denotes the average expression level.

Comparative analysis of cellular proportions revealed clinical state-dependent alteration of the immune landscape (**Figure 2**). CD4^+^ T cell frequencies remained relatively stable (37.3% in BKPyVAN, 38.6% in BKPyV DNAemia, 37.5% in Stable), whereas CD8^+^ T cell and myeloid compartments showed pronounced variation. CD8+ T cells (all subsets combined) were most abundant in the Stable state (26.8%), lower in BKPyV DNAemia (7.8%), and intermediate in BKPyVAN (19.9%). Monocytes were most abundant in BKPyV DNAemia (38.5%), intermediate in BKPyVAN (21.5%), and lowest in the Stable state (7.1%). BKPyV DNAemia was characterized by a high proportion of CD14+ classical monocytes (21.1%) and CD16^+^ non-classical monocytes (13.8%), accompanied by CD4^+^ central memory T cells (12.5%) and macrophages (11.0%). These patterns indicate clinical-state-associated differences in innate and adaptive immune composition across Stable, BKPyV DNAemia, and BKPyVAN.

**Figure 2:**
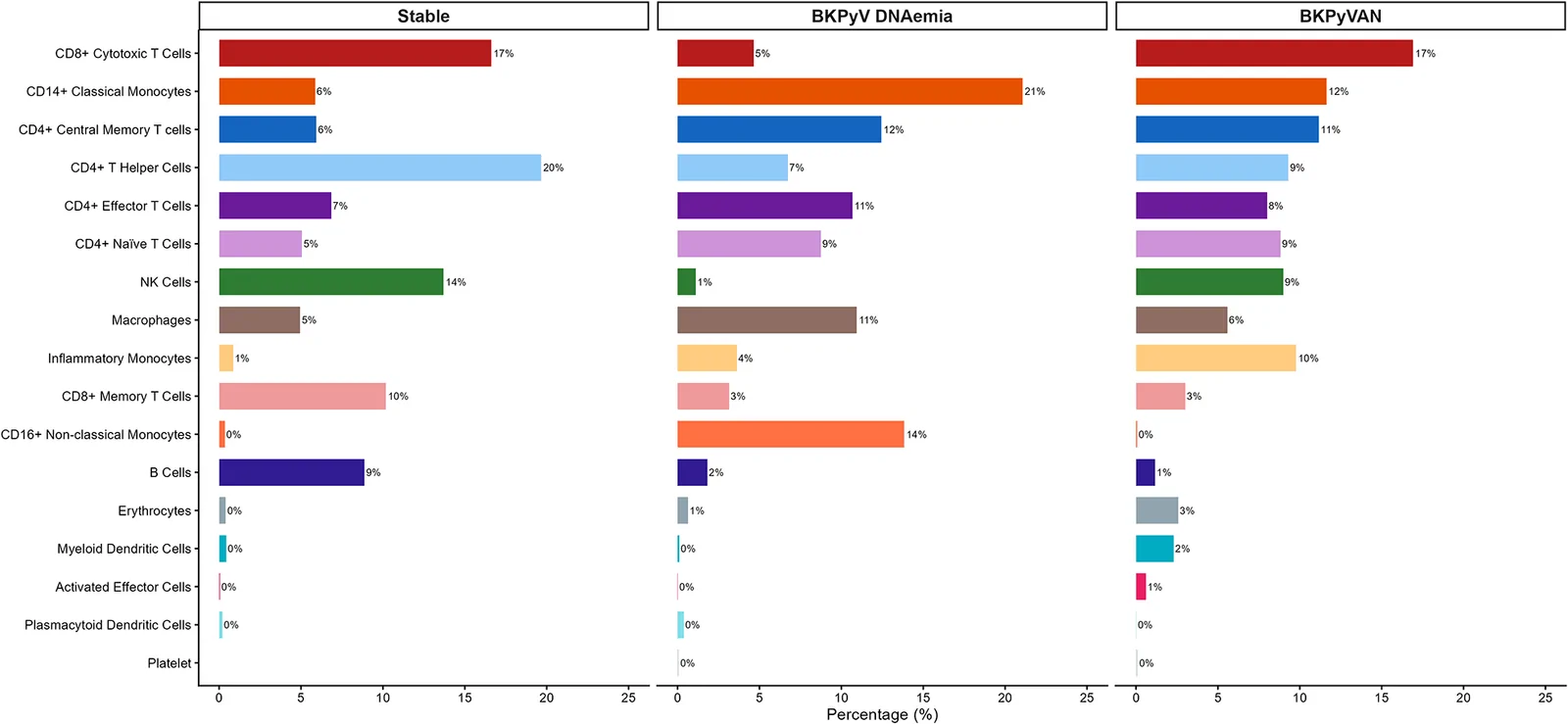
Immune cell composition shifts across clinical conditions. Bar plots showing the relative proportions of immune cell types in Stable, BKPyV DNAemia, and BKPyVAN samples. CD8+ cytotoxic T cells (Cluster 0) were comparably high in the Stable and BKPyVAN groups (17% each) and lower in BKPyV DNAemia (5%). Monocyte subsets, particularly CD14+ classical monocytes (21%) and CD16+ non-classical monocytes (14%), were most abundant in BKPyV DNAemia. Activated Effector Cells (AECs) were more frequent in BKPyVAN (0.6%) than in the other conditions.

### 3.3 Activated effector cells: a rare but transcriptionally distinct population

Beyond compositional changes, we identified a rare but transcriptionally distinct population, termed Activated Effector Cells (AECs). AECs accounted for only 0.60% of cells in the BKPyVAN group (104 of 17,238 cells) yet contributed 12.15% of Bonferroni-significant cluster-associated marker calls, the highest contribution among the 17 clusters (rank 1; **Supplementary Table S4**). This ranking was preserved under a coarser 10-population annotation (16.76%, rank 1). AECs were detected in all three BKPyVAN individuals (33, 48, and 23 cells, respectively). AECs showed marked expression of cell cycle–related genes, including CDC20, HIST1H3G, CCNB2, BIRC5, UBE2C, and CDC25C (**Figure 3**). Lineage-marker analysis showed a lymphoid-associated marker profile with little evidence of myeloid or B-cell marker expression (**Supplementary Figure S5**). AECs showed elevated doublet scores alongside high RNA content and cell-cycle activity; however, lineage-marker assessment and CD3D/CD14 co-expression analysis suggest that the AEC profile is unlikely to be explained solely by classical cross-lineage doublets, although a residual technical contribution cannot be fully excluded (**Supplementary Figures S6**, **S7**). In BKPyVAN samples, AUC-based GSEA comparing AECs with other immune cells showed strong enrichment of cell-cycle, mitotic cell-cycle, E2F-target, DNA-replication, and RNA-processing pathways, consistent with a proliferation-associated transcriptional program. A lineage-restricted sensitivity analysis comparing AECs with CD8+ cytotoxic T cells within BKPyVAN yielded the same dominant proliferation-associated pathways, including cell cycle, E2F targets, and the G2M checkpoint.

**Figure 3:**
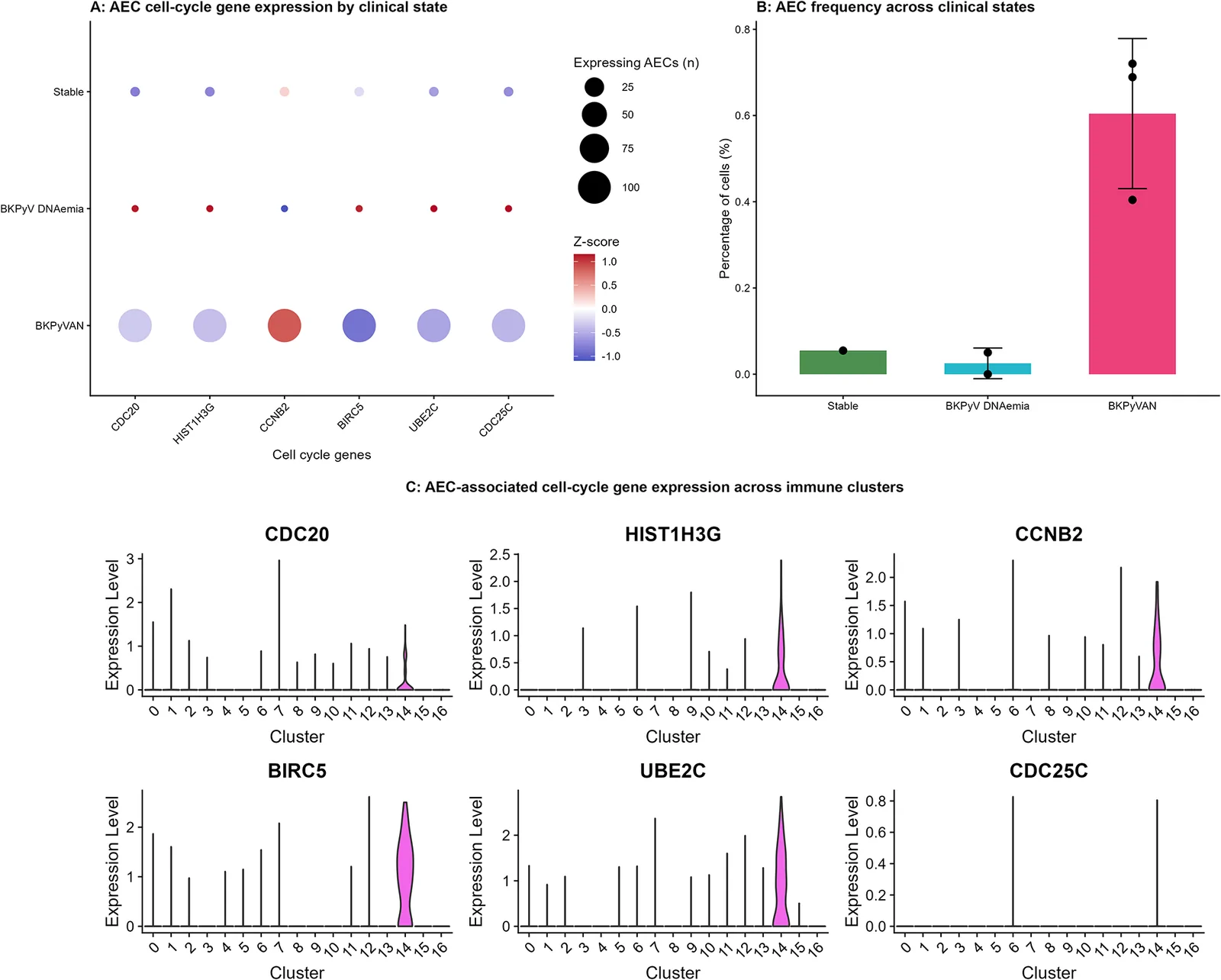
Characterization of activated effector cells (AECs). **(A)** Dot plot of selected cell cycle-related genes (CDC20, HIST1H3G, CCNB2, BIRC5, UBE2C, CDC25C) within AECs across clinical states. Dot size indicates the number of expressing AECs; color represents the Z-scored mean expression. **(B)** Bar plot of AEC frequency across clinical states (mean ± SD; black points indicate individual recipients). AEC frequency was higher in BKPyVAN (0.60%) than in BKPyV DNAemia (0.02%) and Stable (0.05%). **(C)** Violin plots of key cell cycle marker genes showing their high expression in AECs (cluster 14) relative to other immune clusters.

### 3.4 Myeloid cell subsets: clinical-state-associated transcriptional differences

Monocyte subsets differed across clinical conditions (**Figure 4**). Inflammatory monocytes (Cluster 8), defined by high IL1B, CXCL8, and PTGS2 expression, represented only 0.9% of PBMCs in the Stable state and 9.8% in BKPyVAN (**Figure 4A**). Marker expression patterns also differed across clinical states, with IL1B and CXCL8 prominent in BKPyVAN and CCL2 and GPNMB prominent in BKPyV DNAemia (**Figure 4B**).

**Figure 4:**
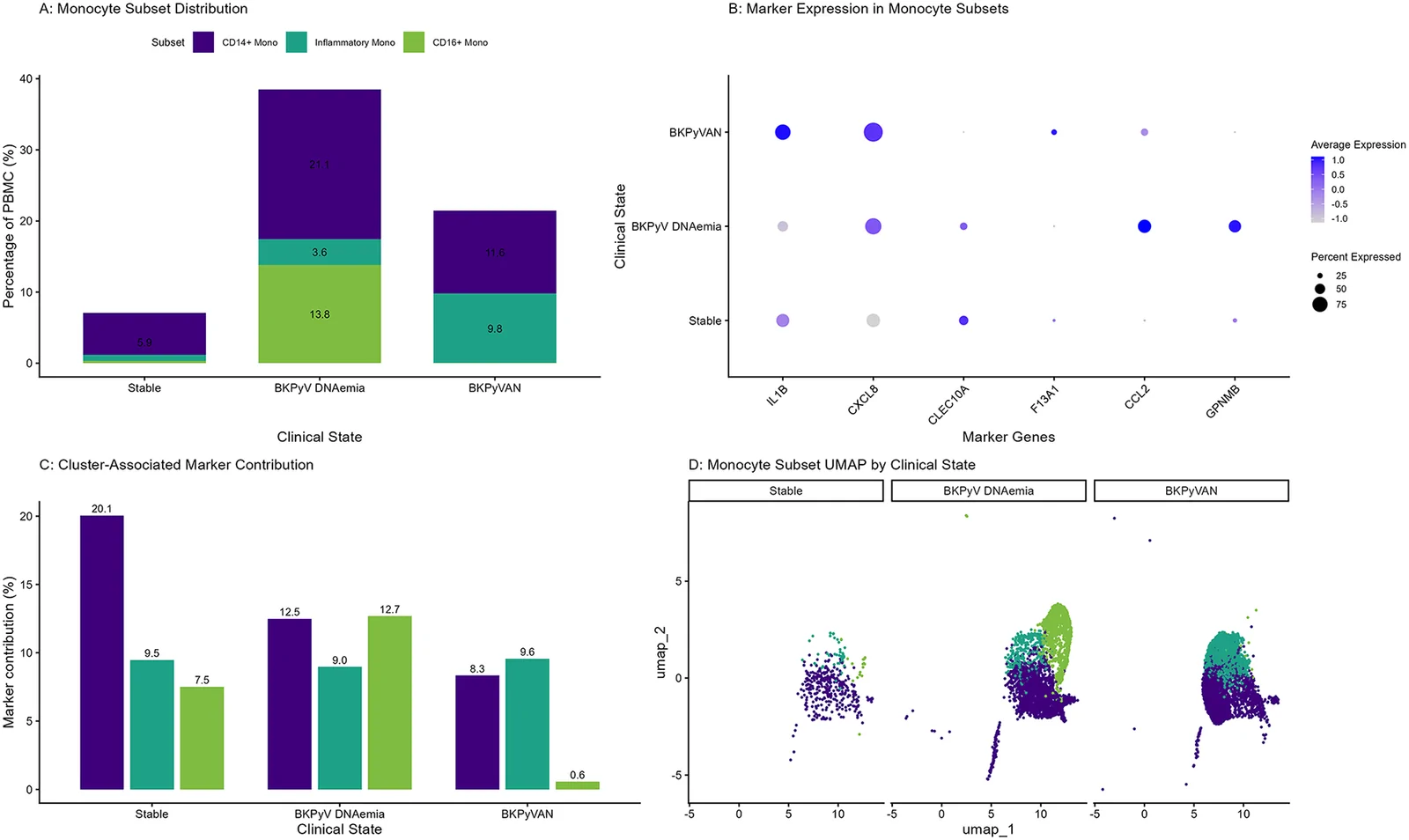
Monocyte subset remodeling and clinical-state-associated transcriptional differences. **(A)** Stacked bar plot showing the relative abundance of CD14+ classical, CD16+ non-classical, and inflammatory monocytes across clinical states. **(B)** Dot plot of inflammatory and functional markers (IL1B, CXCL8, CLEC10A, F13A1, CCL2, GPNMB) across monocyte subsets and clinical states. **(C)** Bar plot of cluster-associated marker contribution (%) for each monocyte subset across clinical states. Among the three monocyte subsets, the highest contribution was observed for CD14+ classical monocytes in Stable, CD16+ non-classical monocytes in BKPyV DNAemia, and inflammatory monocytes in BKPyVAN. **(D)** UMAP projections of monocyte subsets across clinical states, showing the distribution of CD14+ classical, CD16+ non-classical, and inflammatory monocytes within each state.

CD14+ classical monocytes (Cluster 1) were most abundant in BKPyV DNAemia (21.1% of PBMCs) compared with the Stable state (5.9%) and BKPyVAN (11.6%) (**Figure 4A**). CD16+ non-classical monocytes (Cluster 10) were enriched in BKPyV DNAemia (13.8% of cells) and were rare in the Stable state and BKPyVAN (0.3% and 0.1%, respectively). Among monocyte subsets, the leading cluster-associated marker contribution differed across clinical conditions: CD14+ classical monocytes in the Stable state (20.05%), CD16+ non-classical monocytes in BKPyV DNAemia (12.69%), and inflammatory monocytes in BKPyVAN (9.57%) (**Figure 4C**; **Supplementary Table S4**). Monocyte subset distributions across clinical conditions are visualized by UMAP (**Figure 4D**).

### 3.5 Lymphoid populations: functional adaptations across disease states

CD8^+^ cytotoxic T cells (Cluster 0) showed differences in abundance and cytotoxic-marker expression across clinical states. In the Stable state, CD8^+^ T cells showed modest expression of cytotoxic markers including GZMH and CCL5. In BKPyV DNAemia, CD8^+^ T-cell frequency was lower, whereas expression of effector molecules including GZMH, FGFBP2, and FASLG was higher, consistent with an enhanced cytotoxic transcriptional program in BKPyV DNAemia. In BKPyVAN, CD8^+^ cytotoxic cells (Cluster 0) comprised 16.9% of cells and showed high expression of GZMH and IFNG.

NK cells (Cluster 6) were numerically prominent in the Stable state (13.7% of PBMCs) and were lower in frequency in BKPyV DNAemia and BKPyVAN. NK cells expressed cytotoxicity-associated receptors, including KLRC1, across all clinical states, with the highest expression in BKPyV DNAemia.

CD4^+^ naïve T cells (Cluster 5) represented 5.06% of cells in Stable, 8.75% in BKPyV DNAemia, and 8.82% in BKPyVAN.

### 3.6 Pathway-level signatures distinguish disease states

To elucidate the molecular mechanisms underlying clinical-state-associated immune responses, AUC-based GSEA was performed across major immune subsets and disease states (**Figure 5**). Multiple pathways were consistently enriched across lineages, indicating shared core response programs. RNA processing and metabolism pathways (REACTOME_METABOLISM_OF_RNA, GOBP_RNA_PROCESSING) showed strong activation in CD8^+^ T cells, CD14^+^ classical monocytes, CD4^+^ naïve T cells, and inflammatory monocytes in BKPyVAN versus Stable, suggesting that enhanced RNA regulatory capacity is a common feature of antiviral activation. Intracellular transport pathways (GOBP_INTRACELLULAR_TRANSPORT) were broadly enriched across these cell types, highlighting the importance of vesicular trafficking and protein transport in coordinated effector responses (25, 26).

**Figure 5:**
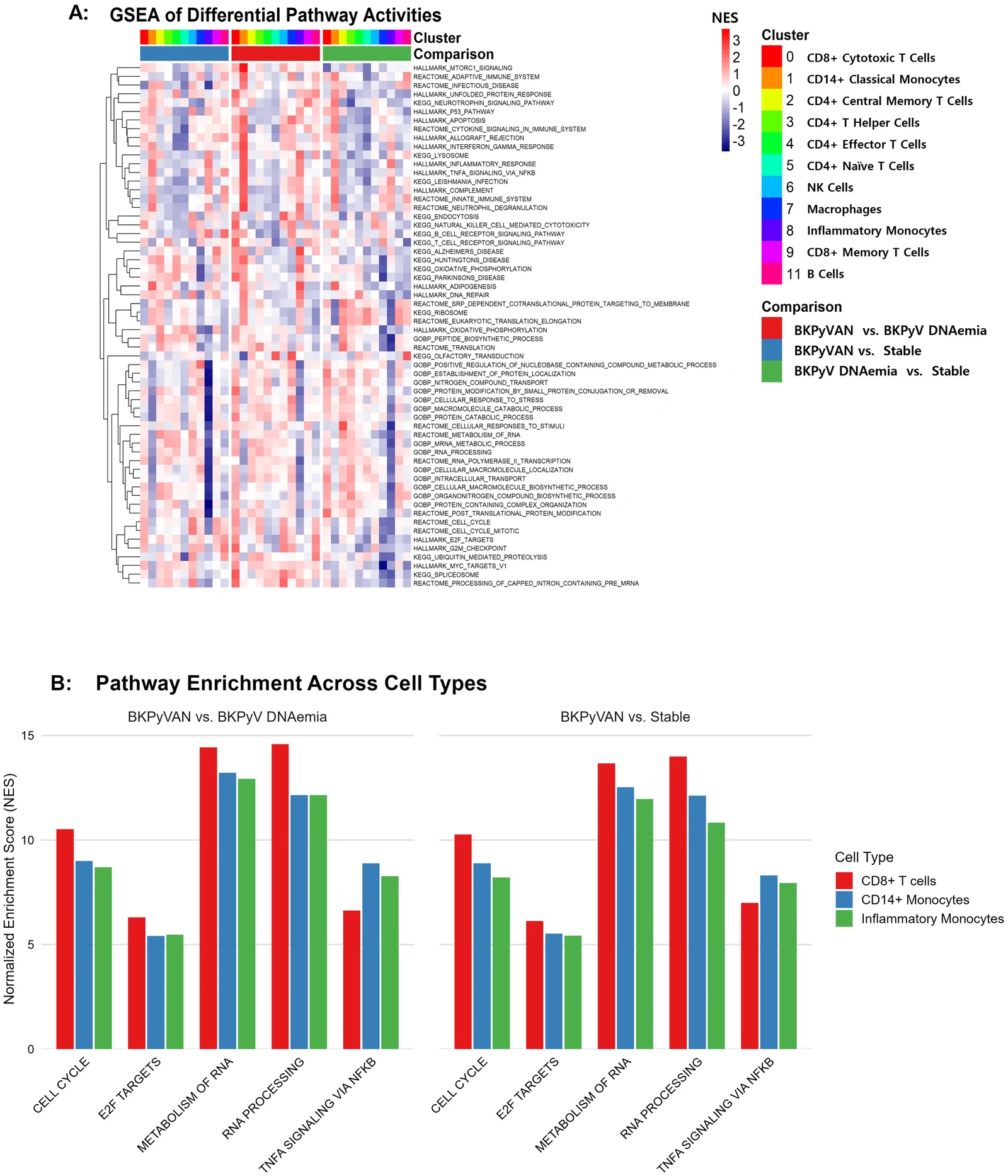
Pathway-level transcriptional changes across immune cell subsets. **(A)** Heatmap of normalized enrichment scores (NES) from GSEA across cell clusters. Comparisons include BKPyVAN vs. BKPyV DNAemia (red), BKPyVAN vs. Stable (blue), and BKPyV DNAemia vs. Stable (green). Rows correspond to pathways; columns represent cell-type clusters (clusters 0–11, as labeled). Clusters with insufficient cell numbers for reliable enrichment analysis were not included. Selected hallmark and curated pathways from MSigDB are shown. **(B)** Bar plots of NES values for five representative pathways across CD8+ T cells, CD14+ monocytes, and inflammatory monocytes. Left panel shows BKPyVAN vs. BKPyV DNAemia; right panel shows BKPyVAN vs. Stable. Only pathways with FDR < 0.05 are displayed. RNA metabolism and cell-cycle pathways showed particularly strong enrichment across these cell types.

Cell type–specific signatures were observed (**Figure 5B**). In BKPyVAN, CD8^+^ T cells showed enrichment of RNA processing and macromolecule biosynthesis compared with Stable and additional cellular response pathways compared with BKPyV DNAemia, consistent with a reprogrammed effector state. CD14^+^ classical monocytes in BKPyVAN demonstrated enrichment of RNA metabolism and innate immune system pathways, including TNF-α signaling via NF-κB, whereas inflammatory monocytes showed strong TNF-α/NF-κB signaling and peptide biosynthesis pathways, in line with their pro-inflammatory phenotype.

### 3.7 Pseudobulk differential expression analysis

To complement single-cell analyses and reduce single-cell-level pseudoreplication, pseudobulk differential expression was performed using DESeq2 with a design including cluster and condition. Comparison between BKPyVAN and BKPyV DNAemia identified 1,825 DEGs, including 1,020 upregulated and 805 downregulated genes. The volcano plot (**Figure 6A**) highlighted upregulated genes such as AC004556.3, CREM, HES4, ZNF331, NR4A2, and MTFP1, and downregulated genes including *FN1, TREM2, RNASE1, MRC1*, and *FPR3*. A heatmap of the top 30 DEGs (**Figure 6B**; **Supplementary Figure S8**) showed distinct condition-associated expression patterns across BKPyVAN, BKPyV DNAemia, and Stable samples, illustrating disease state–specific transcriptional profiles. A cross-method comparison with single-cell differential expression is provided in **Supplementary Figure S9**. In 100 label-permutation analyses, the observed minimum p-value was more extreme than the minima obtained under all permutations (empirical p = 0.0099; **Supplementary Figure S10**).

**Figure 6:**
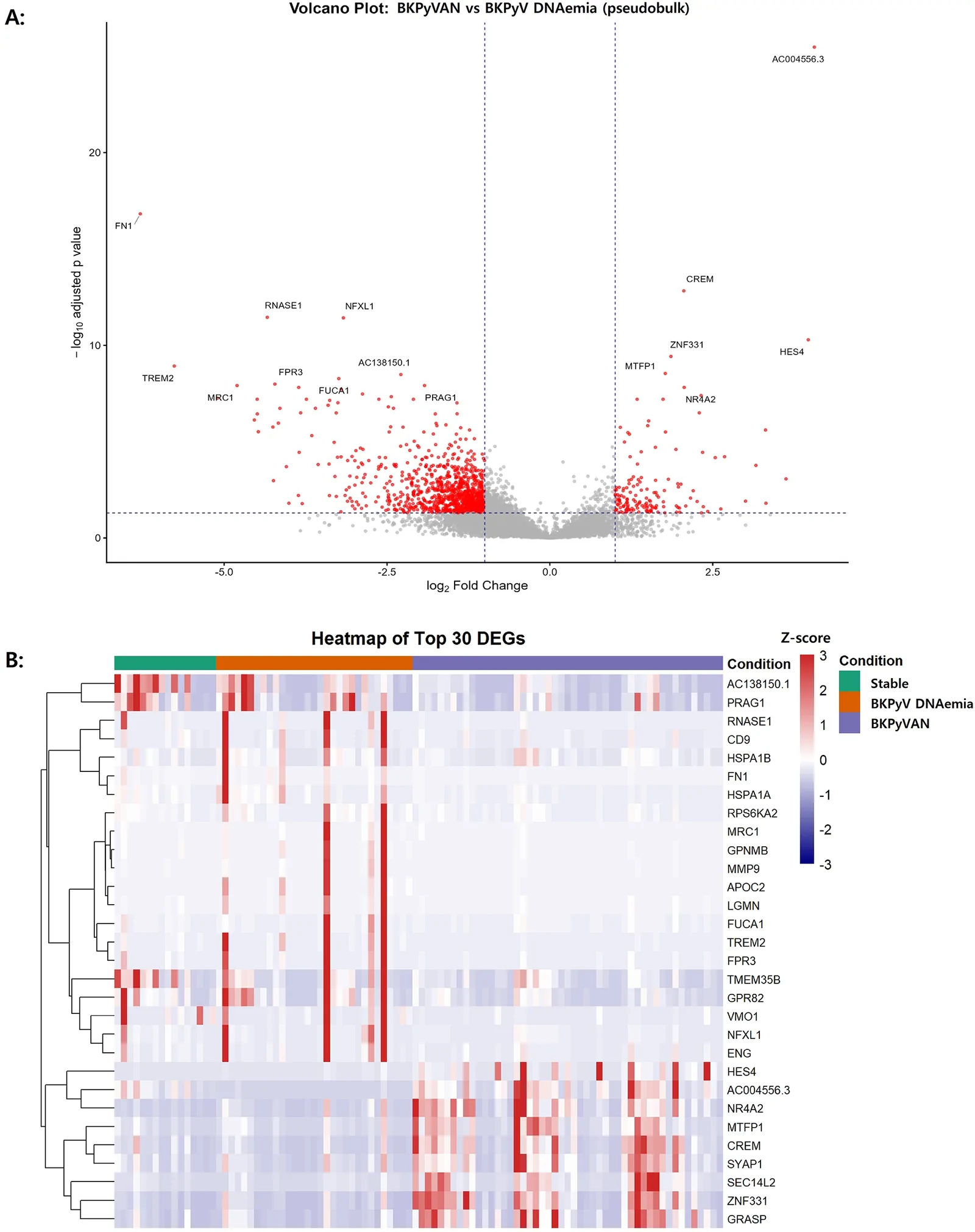
Pseudobulk differential expression reveals distinct disease signatures. **(A)** Volcano plot of log_2_ fold-change vs. –log_10_(adjusted p-value) comparing BKPyVAN vs. BKPyV DNAemia from pseudobulk analysis. Significant genes (red; adjusted p < 0.05 and |log_2_FC| ≥ 1) and non-significant genes (gray) are indicated. Fifteen representative genes are labeled, including upregulated genes (AC004556.3, CREM, HES4, ZNF331, NR4A2, MTFP1) and downregulated genes (FN1, TREM2, RNASE1, MRC1, FPR3). **(B)** Heatmap of the top 30 differentially expressed genes (ranked by adjusted p-value) identified from the condition effect in the DESeq2 model (design: ~ cluster + condition), displayed across 96 patient–cluster pseudobulk profiles. Gene expression values were transformed to Z-scores (row-scaled) and capped at ±3 to highlight expression patterns. Columns represent individual patient–cluster profiles and are grouped by clinical condition (Stable, green; BKPyV DNAemia, orange; BKPyVAN, purple); genes (rows) were grouped by hierarchical clustering.

### 3.8 Candidate transcriptional readouts from leading-edge gene analysis

To prioritize candidate transcriptional readouts for noninvasive monitoring of BKPyV infection, leading-edge gene analysis was applied to significantly enriched pathways. Three transcriptomic signature groups emerged. First, within BKPyVAN, AECs showed strong enrichment of cell-cycle and mitotic pathways, with leading-edge genes including CDK1, CCNB2, CDC20, TOP2A, and AURKB, identifying these as candidate proliferation-associated readouts. Second, inflammatory monocytes displayed TNF-α/NF-κB–related signatures (NFKBIA, TNFAIP3, IL1B, CXCL8) that distinguished BKPyV infection states from Stable conditions. Third, RNA processing genes (*SNRNP200, DDX39B, PRPF8*) enriched across multiple lineages formed a cross-cell-type “RNA activation” signature that may be particularly amenable to blood-based monitoring.

### 3.9 Cell type-specific transcriptional differences across clinical states

Finally, we examined transcriptional differences within individual cell types across clinical states (**Figure 7**). These cell type-resolved patterns complemented the population-level analyses by identifying genes that varied between clinical states within each major lineage. In CD8^+^ cytotoxic T cells, S100B and ENC1 expression was lowest in Stable, intermediate in BKPyV DNAemia, and highest in BKPyVAN (**Figure 7A**). In CD14^+^ classical monocytes, BKPyVAN was associated with lower expression of FN1 and CADM1 (**Figure 7B**). In inflammatory monocytes, TREM2 expression was higher in BKPyV DNAemia and lower in BKPyVAN (**Figure 7C**).

**Figure 7:**
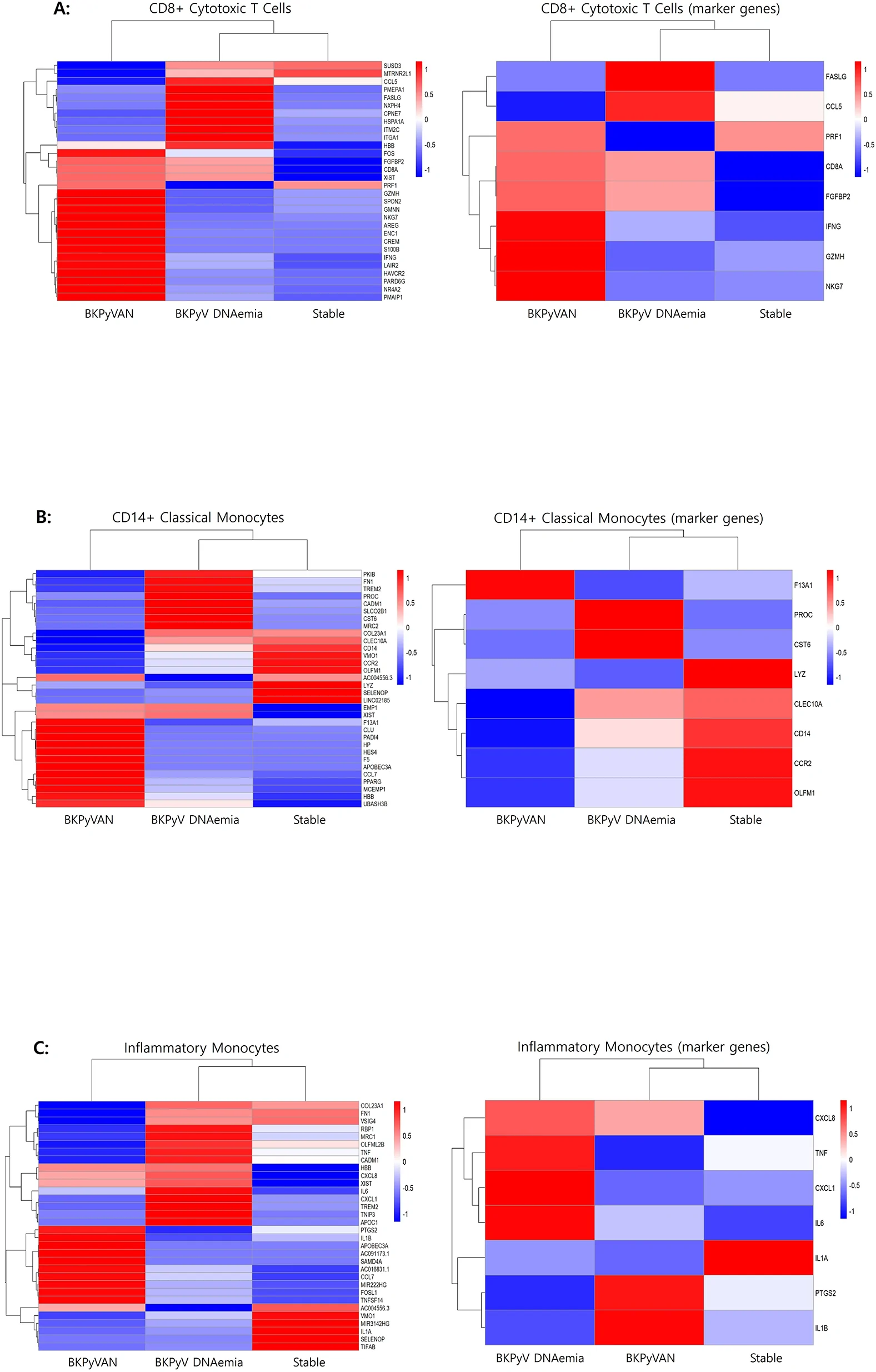
Cell type-specific transcriptional differences across clinical states. Heatmaps showing differentially expressed genes (left panels) and canonical marker genes (right panels) in three key immune cell populations across disease states. In each panel, samples were ordered based on hierarchical clustering to highlight cell type-specific expression patterns, resulting in different sample arrangements across cell types. **(A)** CD8^+^ cytotoxic T cells: Left panel shows a heatmap of Z-score normalized expression for differentially expressed genes, including upregulation of *S100B* and *ENC1* in BKPyVAN compared with Stable condition, with BKPyV DNAemia showing intermediate expression. Right panel displays expression patterns of canonical cytotoxic markers (*CD8A, NKG7, FASLG, CCL5, PRF1, FGFBP2, IFNG, GZMH*) across disease states. **(B)** CD14^+^ classical monocytes: Left panel shows DEG heatmap across clinical groups, highlighting downregulation of *FN1* and *CADM1* in BKPyVAN samples. Right panel displays marker gene expression patterns (*CLEC10A*, *OLFM1*, *F13A1*, *PROC*, *CST6*, *LYZ*, *CD14*, *CCR2*), with distinct expression profiles across disease states. **(C)** Inflammatory monocytes: Left panel shows DEG heatmap across conditions, featuring opposing expression patterns of *TREM2* between BKPyVAN (downregulated) and BKPyV DNAemia (upregulated). Right panel displays inflammatory mediator expression (*CXCL8*, *TNF*, *CXCL1*, *IL6*, *IL1A*, *PTGS2*, *IL1B*), with differential expression patterns across disease states.

## 4 Discussion

This study identified peripheral immune remodeling associated with BK polyomavirus infection and BKPyVAN in kidney transplant recipients. Across three clinical states—stable graft function, BKPyV DNAemia, and biopsy-proven BKPyVAN—distinct shifts in innate and adaptive compartments were observed, and an uncommon but transcriptionally distinct population, termed AECs, emerged as a prominent feature of BKPyVAN in this cohort. These data support a model in which both quantitative and qualitative alterations in peripheral immunity may underlie the differences observed between BKPyV DNAemia and biopsy-proven BKPyVAN.

A key observation is that AECs, although representing only a small fraction of cells in the BKPyVAN group, accounted for a disproportionately large share of cluster-associated marker calls. This finding is in line with the broader concept that rare cellular subsets can harbor distinctive biological programs (27), as shown for ionocytes in the airway epithelium or for highly transcriptionally active fractions of BKPyV-infected renal epithelial cells *in vitro* (28, 29). Recent single-cell studies of BKPyV-infected renal proximal tubular epithelial cells and of BKPyVAN kidney tissue have similarly demonstrated substantial cell-to-cell transcriptional heterogeneity, including altered cell-cycle, stress-response, and epithelial–immune programs (16, 17, 29). Although these renal epithelial and intrarenal observations are biologically distinct from the circulating immune populations analyzed here, they reinforce the value of single-cell approaches for resolving transcriptionally distinct cellular states during BKPyV infection. In our study, AECs—despite comprising only 0.60% of cells in the BKPyVAN group—accounted for 12.15% of cluster-associated marker calls, the highest contribution among the 17 clusters, and this ranking was preserved under a coarser 10-population annotation. While the precise lineage origin of AECs remains to be fully established, AECs exhibited a lymphoid-associated marker profile (**Supplementary Figure S5**) together with a pronounced cell-cycle phenotype (**Supplementary Figures S6**, **S7**). Within-BKPyVAN GSEA further showed enrichment of cell-cycle and RNA-processing pathways, consistent with a proliferation-associated transcriptional program. The prominent cell-cycle program may complicate interpretation of lineage-defining markers. Given the limited number of AECs, particularly outside BKPyVAN, these observations should be regarded as hypothesis generating and warrant validation in larger, longitudinal cohorts.

The behavior of NK cells and monocyte subsets across clinical states provides additional insight into potential surveillance gaps (30). NK-cell frequency was 13.7% in the Stable state and was lower in individuals with BKPyV DNAemia and BKPyVAN. Prior studies have linked altered NK cell phenotypes, including dysregulated killer immunoglobulin-like receptor (KIR) expression and BKPyV-specific NK responses, with increased risk of BKPyV DNAemia and BKPyVAN (31). In this context, the reduction in NK cell abundance in BKPyVAN may reflect inadequate innate surveillance and cytotoxic function, potentially contributing to viral persistence and tissue invasion despite preserved expression of cytotoxicity receptors.

Beyond NK cells, monocyte subsets also differed across clinical states. Early innate immune activation can shape subsequent adaptive antiviral immunity (32, 33). Although monocytes have not been directly implicated in BKPyV-specific immunity, their clinical-state-associated remodeling may reflect, rather than drive, the systemic inflammatory response to tissue-invasive viral disease, potentially shaping antiviral immunity through antigen presentation, cytokine production, and support of cytotoxic responses. A BKPyV-specific monocyte role, however, cannot be established from these data. The higher proportion of inflammatory monocytes in BKPyVAN may reflect an altered inflammatory state associated with BKPyVAN. The distinct patterns of monocyte subsets across clinical states, in which CD16^+^ non-classical and CD14^+^ classical monocytes were prominent in BKPyV DNAemia and inflammatory monocytes were more abundant in BKPyVAN, suggest distinct innate involvement across clinical states. Adaptive immune responses also differed markedly among the three clinical states. BK polyomavirus-specific T cell responses are critical for viral control, and their absence correlates with persistent BKPyV DNAemia (8); consistent with this, biopsy-proven BKPyVAN has been associated with impaired BKPyV-specific CD4^+^ and CD8^+^ T-cell proliferation, cytokine secretion, and cytotoxic function, with allogeneic CD4 help capable of sustaining CD8 responses (10). In our cohort, CD8^+^ T-cell frequency was higher in BKPyVAN than in BKPyV DNAemia, with high cytotoxic gene expression; however, these features were observed despite established BKPyVAN and therefore do not necessarily indicate effective viral control. The lower CD8^+^ T-cell proportion in BKPyV DNAemia should also be interpreted in light of the compositional nature of single-cell data, which reflect relative rather than absolute frequencies; the concurrent expansion of monocyte compartments (CD14^+^, 21.1%; CD16^+^, 13.8%) may contribute to a lower relative proportion of CD8^+^ T cells. BKPyV-specific CD8^+^ T cells may also represent a minor subset not individually resolved in unsorted profiling. Given only two BKPyV DNAemia individuals, these interpretations remain descriptive. Together, these observations support a model in which both innate (NK and monocyte subsets) and adaptive (CD8^+^ T cells) arms exhibit clinical-state-associated imbalances, creating windows of vulnerability that can be conceptualized as surveillance gaps across the BKPyV disease spectrum.

Pathway-level analysis using AUC-based GSEA provided additional insights that extend beyond single-gene markers. Broad enrichment of RNA metabolism and RNA processing pathways across CD8^+^ T cells, monocyte subsets, and CD4^+^ naïve T cells in BKPyVAN individuals suggests shared enhancement of RNA handling and translational programs across these immune populations.

In monocytes, TNF-α/NF-κB signaling pathways differentiated individuals with BKPyVAN and BKPyV DNAemia from those with stable graft function, consistent with prior reports implicating inflammatory pathways and TNF signaling in BKPyV-induced cellular reprogramming. The distinct proliferation-associated signature of AECs further highlights the prominence of cell-cycle programs in BKPyVAN, raising the possibility that pathway-level analysis can reveal biologically relevant immune signatures even in contexts of significant cellular heterogeneity (34).

For potential clinical translation, our findings suggest that PCR-based panels targeting leading edge genes (e.g., *CDC20*, *AURKB*, *BIRC5* for AECs; *IL1B*, *CXCL8* for inflammatory monocytes; *APOC1*, *CCL2* for CD16^+^ monocytes) might complement BKPyV DNA load testing in risk stratification. Such panels could be integrated with existing BKPyV DNA testing and BKPyV-specific T cell assays to refine timing and intensity of immunosuppression reduction or adjunctive therapies. Ultimately, incorporation of cell type– and pathway-specific transcriptional readouts into peripheral blood assays may enable earlier detection of high-risk immune states before overt nephropathy develops (35). These concepts remain hypothesis-generating and will require extensive validation before clinical implementation.

Although our analysis was restricted to the peripheral compartment, a recent single-cell study of BKPyV-infected kidney allografts (Marvin et al.) independently reported increased RNA-metabolic and proliferative programs across innate immune populations, together with cytokine- and TNF-related responses in NK cells and altered monocyte signaling (36). Despite the distinct tissue compartment, this convergence with several pathways identified in our peripheral data supports further investigation of potentially coordinated systemic and graft-localized immune remodeling during BKPyV infection.

This study has several limitations. The small sample size (n = 6) and single-center design limit statistical generalizability, although the use of complementary single-cell, pseudobulk, and permutation-based analyses provides additional support for the internal consistency of the findings within this cohort. Annotated cluster identities were corroborated by feature plots of canonical lineage markers (**Supplementary Figure S11**), and immune cell composition varied among individuals across the cohort, including within the BKPyV DNAemia and BKPyVAN groups (**Supplementary Figure S12**), highlighting inter-individual heterogeneity that warrants cautious interpretation of group-level comparisons. Limitations inherent to scRNA-seq, including dropout and low mRNA capture efficiency, were addressed in part through complementary single-cell, pseudobulk, and pathway-level analyses. Peripheral immune profiles may not fully reflect intra-renal immune dynamics. Future work integrating matched blood and kidney single-cell data will be important to distinguish systemic from tissue-restricted responses. Finally, statistical power for AEC comparisons was limited by their rarity in non-BKPyVAN conditions, and larger and longitudinal cohorts are needed for definitive characterization. Although AECs were detected in all three BKPyVAN individuals, the cohort did not include a BKPyV-negative nephropathy control group; therefore, the specificity of this and other peripheral immune features to BKPyVAN, as opposed to nephropathy more broadly, cannot be established here and warrants future study. Immunosuppressive regimens differed among participants, and although all recipients were CMV-seropositive donor/recipient pairs without active CMV or other concurrent infection at sampling (**Supplementary Table S5**), the effects of BKPyV replication, BKPyVAN, and immunosuppressive modification on peripheral immune-cell profiles could not be fully disentangled in this small cohort. Because samples were collected cross-sectionally from different recipients at different post-transplant time points, the observed differences across clinical states should not be interpreted as within-patient temporal trajectories.

Our exploratory single-cell analysis provides insights into peripheral immune signatures across the clinical spectrum of BK polyomavirus infection in kidney transplant recipients. The observation of AECs as a rare but transcriptionally distinct population with a marked proliferation-associated program in BKPyVAN individuals in this cohort suggests that numerical representation alone may be insufficient to capture immune components of potential biological importance. Clinical-state-associated remodeling of NK cells, monocyte subsets, and CD8^+^ T cells, together with convergent pathway-level signatures, supports a model of surveillance gaps arising from imbalances in both innate and adaptive immunity. These findings identify peripheral transcriptomic features—including the rare AEC population and cell cycle, TNF-α/NF-κB, and RNA-processing signatures—as candidates for further evaluation in BKPyV-associated risk stratification and monitoring.

## Data Availability

The data presented in the study are deposited in the NCBI Gene Expression Omnibus (GEO) repository, accession number GSE295129, and in the NCBI Sequence Read Archive (SRA) under BioProject accession number PRJNA1246980.
