# Supplementary material for: Single-cell profiling of peripheral immune remodeling in BK polyomavirus–associated nephropathy after kidney transplantation
Source: Front Immunol. 2026 Oct 1;17:1939071. doi: 10.3389/fimmu.2026.1939071 (PMC13668843; doi:10.3389/fimmu.2026.1939071)
Supplement: Supplementary file 1 [file DataSheet1.docx]

**Supplementary Information**

# 1 Supplementary Materials and Methods

## 1.1 PBMC Preparation and Sequencing

Cryopreserved PBMCs were thawed at 37°C in DMEM supplemented with 10% fetal bovine serum. After two washes with cold 0.04% BSA in calcium- and magnesium-free PBS (centrifuged at 300g for 5 minutes at 4°C), cells were gently resuspended in chilled Stain Buffer (BD Biosciences, catalog #554656). Cell viability and counts were determined using the LUNA-FX7™ Automated Fluorescence Cell Counter (Logos Biosystems) with AO/PI staining. Samples were labeled with Cell Multiplexing Oligos (CMOs) according to the 10x Genomics protocol (document CG000391, product #1000261). For each sample, 1 × 10^6^ cells were resuspended in 100 µl of CMO solution and incubated for 5 minutes at room temperature. After incubation, cells were washed three times with chilled buffer containing 1% BSA in PBS (Ca^2+^ and Mg^2+^ free) and resuspended to a final concentration of 1,300–1,600 cells/μl. Three samples, each with a unique CMO tag, were pooled per multiplexing batch; two multiplexing batches were used to process all six samples. Specific CMO assignments for each sample and run are detailed in the accompanying GEO record (GSE295129).

Single-cell RNA and CMO libraries were prepared using the Chromium Single Cell 3′ v3.1 platform (10x Genomics), following manufacturer protocols. Sequencing was performed on an Illumina HiSeqXten system, generating 150 bp paired-end reads ^1^. Libraries were sequenced to an approximate depth of 30,000 read pairs per cell for the gene expression library and 5,000 read pairs per cell for the Cell Multiplexing library.

## 1.2 Data Processing and Quality Control

Initial quality control was conducted using FastQC. Raw sequencing data were processed using Cell Ranger (v6.0.2; 10x Genomics) with the GRCh38 reference transcriptome (10x Genomics, version 2020-A). The *cellranger multi* pipeline was used to demultiplex samples using CMO barcodes and generate UMI-based gene expression matrices for each sample. Downstream single-cell preprocessing and clustering were performed in R using Seurat, with additional packages used for differential-expression and pathway analyses. Sequencing was performed in two multiplexing batches, each containing three samples. For one batch (Stable, BKPyV DNAemia_1, and BKPyVAN_1), two independent sequencing runs were performed, and reads from both runs were merged for analysis. The second batch (BKPyV DNAemia_2, BKPyVAN_2, and BKPyVAN_3) was sequenced once.

In total, we obtained 31,779 high-quality cells across all samples. Cell recovery varied by clinical group: 5,473 cells from the stable allograft patient, 9,068 cells from two BKPyV DNAemia patients (3,976 from BKPyV DNAemia_1, 5,092 from BKPyV DNAemia_2), and 17,238 cells from three BKPyVAN patients (4,582 from BKPyVAN_1, 5,689 from BKPyVAN_2, and 6,967 from BKPyVAN_3). Sequencing quality was high across all samples, with median reads per cell ranging from 19,387 to 27,623. Gene detection rates were consistent, with median genes per cell between 1,338 and 1,525 across samples. Between 62.89% and 69.70% of reads were confidently mapped to the transcriptome, with sequencing saturation of 67.29–74.00% (Supplementary Figure S1). Detailed sequencing metrics for all samples, including mapping statistics, multiplexing efficiency, and sequencing quality scores, are provided in Supplementary Table S1.

## 1.3 Analysis of Single-Cell RNA Sequencing Data

Raw UMI count matrices were loaded into Seurat (v5.1.0) for downstream analysis. Initial filtering was applied to remove low-quality cells and potential doublets. Each sample was normalized and variance-stabilized using the SCTransform method, with glmGamPoi selected to account for sparse gene expression. Given that the samples were processed in two separate multiplexing batches, we implemented an integration strategy to mitigate potential batch effects. To reduce batch effects across runs, we selected 3,000 integration features using *SelectIntegrationFeatures*, followed by anchor identification via reciprocal PCA (*FindIntegrationAnchors*). The final integrated dataset was constructed using the *IntegrateData* function. Integration quality was assessed by examining sample mixing across clusters and proportional contribution of each sample to individual clusters (Supplementary Figure S2).

Dimensionality reduction was performed via principal component analysis (PCA), and two-dimensional embeddings were generated using UMAP. Clustering was performed with a shared nearest neighbor (SNN) graph-based method. Cell type annotation was conducted through analysis of cluster marker genes and comparison with established reference datasets including the Human Cell Atlas immune reference and Monaco et al. PBMC dataset ^2^.

We identified CD8^+^ cytotoxic T cells (cluster 0) based on expression of *CD8A*, *GZMH*, *CCL5*, and *IFNG*; CD4+ T-cell subsets by markers including CD40LG, TNFRSF4, CCR6, CCR7, SELL, TCF7, and LEF1; B cells by *MS4A1*, *CD19*, and *CD79A* expression; NK cells (cluster 6) by *KLRC1*, *KLRF1*, and *NCAM1*; and myeloid populations including classical monocytes (cluster 1, *CD14*, *LYZ*), non-classical monocytes (cluster 10, *FCGR3A*, *APOE*, *APOC1*), and inflammatory monocytes (cluster 8, *IL1B*, *CXCL8*, *PTGS2*). The rare Activated Effector Cells (AECs; cluster 14) were characterized by marked expression of cell-cycle regulators including HIST1H3G, CDC25C, UBE2C, and BIRC5. Clusters were visualized using UMAP embeddings ^3^.

## 1.4 Cluster-Associated Marker Contribution

As a descriptive metric, we quantified the proportion of significant cluster-associated marker calls attributable to each cell cluster separately within each clinical condition (Stable, BKPyV DNAemia, and BKPyVAN). Within each condition, each cluster was compared with all other cells in that condition using FindAllMarkers (Seurat) on the RNA assay with the Wilcoxon rank-sum test (only.pos = FALSE, min.pct = 0.1, |log2FC| ≥ 0.25, min.cells.group = 1), and marker calls with a Bonferroni-adjusted P < 0.05 were retained. Clusters absent from a given condition were not analyzed. For each cluster, the cluster-associated marker contribution was calculated as:

Cluster-associated marker contribution (%) = (number of Bonferroni-significant marker calls for a given cluster / total number of Bonferroni-significant cluster-associated marker calls across all clusters within that condition) × 100

Because the same gene can meet the significance criterion for more than one cluster, the denominator represents the sum of cluster-associated marker calls rather than a set of unique genes. To assess sensitivity to annotation granularity, the analysis was repeated within BKPyVAN using a coarser 10-population annotation in which related CD4 T-cell, CD8 T-cell, monocyte, and dendritic-cell subsets were merged, while AECs were retained as an independent population; all other analytical parameters were unchanged (Supplementary Table S4C).

## 1.5 Disease State-Specific Differential Expression Analysis

As a separate analysis, distinct from the within-condition cluster-marker analysis described above, we examined transcriptional differences across clinical states within each cell type. The dataset was subset by cell type, clinical condition was used as the grouping variable, and differential expression was assessed using FindAllMarkers (Seurat) with the MAST test (only.pos = FALSE, min.pct = 0.1, |log2FC| ≥ 0.25, min.cells.group = 1). Genes with Bonferroni-adjusted P < 0.05 were considered significant. For visualization, AverageExpression was used to calculate mean expression across clinical conditions, followed by row scaling and hierarchical clustering. Because AECs comprised only 3 cells in Stable and 2 cells in BKPyV DNAemia, condition-wise AEC differential-expression results were considered statistically underpowered and were treated as exploratory.

## 1.6 AUC-Based GSEA Analysis for Single-Cell RNA-seq Data

To investigate pathway-level changes, we used an AUC-based gene-ranking approach. Gene Set Enrichment Analysis (GSEA) was performed using the fgsea package in R (version 1.22.0), applying standard scoring parameters (scoreType = "std") with 100,000 permutations to establish statistical significance. For the original clinical-state comparisons, gene ranking used area-under-the-curve (AUC) metrics calculated by the wilcoxauc function from the presto package, measuring the discriminatory power of each gene between the groups in each specified comparison. The resulting AUC values, ranging from 0 to 1, were used as the gene ranking statistic for fgsea, where AUC > 0.5 indicates higher and AUC < 0.5 indicates lower expression in the test condition. This bidirectional ranking, combined with scoreType = "std", captures both up- and down-regulated pathway programs across disease states. Normalized enrichment scores (NES) ranged approximately from −5 to +15, and their distribution is shown in Supplementary Figure S3. For AEC-specific analyses, gene rankings were generated independently of the differential-expression analyses described above; no DEG significance threshold was used as input to GSEA. Within BKPyVAN, AECs (n = 104) were first compared with all other immune cells using genome-wide AUC-based gene rankings (AUC values computed with the ROC test in Seurat), followed by fgsea. As a lineage-restricted sensitivity analysis, AECs were additionally compared with CD8+ cytotoxic T cells within BKPyVAN using the same AUC-ranked GSEA framework. The AEC-specific analyses used MSigDB release 2026.1.Hs.

For the original clinical-state GSEA analyses, we selected four curated collections from MSigDB (version 7.5.1) to focus pathway interpretation on the most relevant biological mechanisms: Hallmark gene sets (n=50) representing coherent biological states or processes; Reactome database pathways (n=1,672) with detailed molecular reactions; KEGG pathways (n=186) covering a broad range of biological systems; and Gene Ontology Biological Process (GO:BP) terms (n=7,961) describing biological objectives to which genes contribute. We applied gene set size filtering (15 ≤ size ≤ 500) to focus on interpretable pathways while excluding overly broad or narrow terms. These pathway sets were prioritized for their relevance to immune function, inflammation, and viral pathogenesis in the transplant setting. GSEA was performed across the complete MSigDB collection, and reporting focused on these four collections.

## 1.7 Pseudobulk Differential-Expression Analysis

To improve statistical power and account for biological variability, we performed pseudobulk differential gene expression analysis by aggregating UMI counts per patient and per Seurat cluster using Seurat (v5.1.0) (*AggregateExpression*, slot = “counts”). The resulting pseudobulk count matrix was analyzed in DESeq2 v1.42.0 with the design formula ∼ cluster + condition, where *condition* included three clinical states: Stable, BKPyV DNAemia, and BKPyVAN. Size factors were estimated using *estimateSizeFactors*, and dispersion was modeled with *estimateDispersions* (fitType = “local”). Wald tests were applied to the contrast BKPyVAN vs. BKPyV DNAemia. Five genes failed to converge during beta estimation and were excluded from downstream analysis. Genes with Benjamini–Hochberg–adjusted p-values (FDR < 0.05) were considered differentially expressed, resulting in 1,825 significant genes (see Supplementary Table S2 for full results).

## 1.8 Permutation Test for Minimum p-value Significance

To evaluate whether the extremely low minimum p-value observed in the pseudobulk analysis could have occurred by chance due to sample size limitations or multiple testing, we performed a label permutation test (n = 100) using DESeq2. In each iteration, condition labels were randomly shuffled, and the Wald test was re-run to identify the minimum raw p-value across all genes.

The empirical p-value was calculated as:

*p*_emp_ = (#{min *p*_perm_ ≤ min *p*_obs_} + 1) / (100 + 1) ≈ 0.0099

The minimum observed p-value in the original analysis was 8.21 × 10^−61^, while permuted minima ranged from 1.52 × 10^−11^ to 3.32 × 10^−3^. The observed minimum was more extreme than the minimum p-values obtained in all 100 permuted-label analyses (Supplementary Table S3).

# 2 Supplementary References

1. 10x Genomics. Chromium Next GEM Single Cell 3' Reagent Kits v3.1 User Guide. Pleasanton, CA: 10x Genomics (2021).

2. Monaco G, Lee B, Xu W, Mustafah S, Hwang YY, Carré C, et al. RNA-Seq Signatures Normalized by mRNA Abundance Allow Absolute Deconvolution of Human Immune Cell Types. Cell Rep. (2019) 26(6):1627-1640.e7. doi: 10.1016/j.celrep.2019.01.041

3. McInnes L, Healy J, Melville J. UMAP: Uniform Manifold Approximation and Projection for Dimension Reduction. arXiv [preprint]. (2018). Available at: https://arxiv.org/abs/1802.03426

**Supplementary Table S1. Detailed Single-Cell RNA-seq Metrics by Sample.**

Comprehensive sequencing quality metrics for each sample across conditions. Includes parameters such as sequencing saturation (67.29–74.00%), Q30 base call percentage, barcode and read quality distributions, and cell filtering thresholds.

The complete table is provided as a separate Excel file (Supplementary_Table_S1.xlsx).

**Supplementary Table S2. Differentially Expressed Genes Between BKPyVAN and BKPyV DNAemia.**

Complete results of pseudobulk differential expression analysis comparing BKPyVAN vs. BKPyV DNAemia samples. The table contains 1,825 significantly differentially expressed genes (FDR < 0.05), including 1,020 upregulated and 805 downregulated genes in BKPyVAN relative to BKPyV DNAemia. For each gene, values include base mean expression, log_2_ fold change, standard error, test statistic, raw p-values, and Benjamini-Hochberg-adjusted p-values.

The complete table is provided as a separate Excel file (Supplementary_Table_S2.xlsx).

**Supplementary Table S3. Permutation Testing for Statistical Robustness.**

Results from the permutation testing framework. Part A contains minimum p-values from 100 random label permutations (range: 1.52 × 10^⁻¹¹^ to 3.32 × 10^⁻³^). The observed minimum p-value (8.21 × 10^⁻⁶¹^) yielded an empirical p-value of 0.0099. Part B provides summary statistics of the minimum p-values from the permutation test, including minimum, quartiles, median, mean, and maximum values**.**

The complete table is provided as a separate Excel file (Supplementary_Table_S3.xlsx).

**Supplementary Table S4. Cell-Type Abundance and Cluster-Associated Marker Contribution by Clinical Condition.**

Part A presents cell counts and frequencies for each of the 17 immune cell clusters across the Stable, BKPyV DNAemia, and BKPyVAN conditions. Part B presents the number and percentage of cluster-associated marker calls per cluster within each condition (each cluster compared with all other cells of the same condition; Wilcoxon rank-sum test, |log2FC| ≥ 0.25, Bonferroni-adjusted P < 0.05), together with the within-condition rank. Because a gene can be a significant marker in more than one cluster, these values represent cluster-associated marker calls rather than unique genes. Part C presents the granularity sensitivity of the AEC marker contribution within BKPyVAN, comparing the 17-cluster annotation with a coarser 10-population annotation in which related CD4 T-cell, CD8 T-cell, monocyte, and dendritic-cell subsets were merged while AECs were retained as an independent population.

The complete table is provided as a separate Excel file (Supplementary_Table_S4.xlsx).

**Supplementary Table S5. CMV Serostatus, Concurrent Infection, Immunosuppressive Regimen at PBMC Sampling, and Overall BKPyV DNAemia Duration.**

Clinical information on CMV donor/recipient serostatus, active CMV infection at PBMC sampling, other documented concurrent infection, immunosuppressive regimen at sampling, and overall documented duration of BKPyV DNAemia for each patient. The overall DNAemia duration may include post-sampling follow-up and was not used as an explanatory variable in the transcriptomic analyses. The complete table is provided as a separate Excel file (Supplementary_Table_S5.xlsx).

**Supplementary Figure S1. Quality control metrics for single-cell RNA-seq data.**

Violin plots showing the distribution of quality control metrics across all six samples: number of detected genes per cell (nFeature_RNA), total UMI counts per cell (nCount_RNA), and percentage of mitochondrial gene expression (percent.mt). Cells passing quality thresholds were retained for downstream analysis.

**Supplementary Figure S2. Assessment of data integration quality.**

(A) UMAP projection of the integrated single-cell data colored by sample, showing that cells from all samples are intermixed across clusters after reciprocal PCA-based integration. (B) Proportional contribution of each sample to each cluster.

**Supplementary Figure S3. Distribution of NES values from AUC-based GSEA.**

Histogram of normalized enrichment scores (NES) from the AUC-based GSEA. Most values are concentrated between approximately −2 and +5, with a sparse upper tail extending to approximately +15; the high NES values reported for individual pathways (up to 14.3 in Figure 5B) fall within this upper tail.

**Supplementary Figure S4. UMAP projections split by clinical condition.**

UMAP embeddings of all cells split by clinical condition (BKPyVAN, BKPyV DNAemia, and Stable). Each panel uses the same embedding coordinates and shows the distribution of annotated immune cell populations within each clinical condition.

**Supplementary Figure S5. Lineage marker expression in Activated Effector Cells.**

Dot plot showing expression of T cell, NK cell, myeloid, and B cell lineage markers, together with selected cell cycle genes, across all immune clusters. Dot size represents the percentage of cells expressing each gene, and color intensity represents average expression level. The Activated Effector Cells (AECs, cluster 14) are included among the clusters shown.

**Supplementary Figure S6. Doublet assessment of AEC cluster.**

(A) Scatter plot of doublet score versus UMI count (nCount_RNA) per cell. AECs (cluster 14) are highlighted in red; other clusters are shown in gray. (B) Scatter plot of doublet score versus gene count (nFeature_RNA) per cell, colored as in (A). (C) Co-expression of CD3D (T-cell marker, x-axis) and CD14 (monocyte marker, y-axis) at single-cell resolution, colored by cell type. The upper-right quadrant indicates cells co-expressing CD3D and CD14. (D) Bar plot of the percentage of cells expressing CD3D only (blue), CD14 only (orange), or both markers (red, potential doublet) across selected clusters.

**Supplementary Figure S7. Cell cycle phase distribution across immune cell clusters.**

(A) Bar plot showing the proportion of cells in G1, S, and G2/M phases for selected immune cell clusters. AECs (Activated Effector Cells) were predominantly assigned to S and G2/M phases based on cell-cycle scoring using canonical S-phase and G2/M-phase gene sets. (B) Scatter plot of G2M cell cycle score versus doublet score per cell. AECs (red) are highlighted; other cells are shown in gray. (C) Bar plot showing the percentage of cells expressing CD14 (myeloid marker, orange) and CD3D (T-cell marker, blue) across selected lymphoid clusters and AECs. (D) Violin plot of CD3E expression across selected lymphoid clusters and AECs.

**Supplementary Figure S8. Alternative visualization of top DEGs with narrower color scale.**

Heatmap of Z-score-normalized expression (capped at ±2) for the top 30 DEGs across 96 patient–cluster pseudobulk profiles.

**Supplementary Figure S9. Cross-method comparison of differentially expressed genes between pseudobulk and single-cell analyses.**

Venn diagram comparing single-cell (MAST; Bonferroni-adjusted P < 0.05) and pseudobulk (DESeq2; Benjamini–Hochberg FDR < 0.05) differential expression analyses for the BKPyVAN vs. BKPyV DNAemia comparison. Of 1,825 pseudobulk DEGs, 657 (36.0%) were also identified in the single-cell analysis, while 1,168 pseudobulk-specific and 2,286 single-cell-specific genes were identified. Nine of the 11 biomarker candidates highlighted in Figure 6A were identified by both methods.

**Supplementary Figure S10. Distribution of minimum p-values from 100 permutations.**

Histogram of minimum p-values from 100 permutations of sample labels. The red vertical line marks the observed minimum p-value (8.21×10⁻⁶¹), which was more extreme than the minimum p-values obtained in all 100 permuted-label analyses; the empirical p-value was 0.0099.

**Supplementary Figure S11. Feature plots of canonical lineage markers.**

Expression of canonical population markers (CD3D, CD8A, CD4, CCR7, SELL, TCF7, NKG7, CD14, FCGR3A, MS4A1, LILRA4, MKI67) projected onto the UMAP embedding, supporting the identities of the annotated immune cell clusters. Color intensity indicates normalized expression level.

**Supplementary Figure S12. Per-individual immune cell composition.**

Relative proportions of annotated immune cell types shown for each of the six samples individually (Stable; BKPyV DNAemia_1–2; BKPyVAN_1–3), corresponding to the composition in Figure 2 split by individual sample. Composition varied among individuals across the cohort, including within the BKPyV DNAemia and BKPyVAN groups, illustrating inter-individual heterogeneity. Cell-type colors are as in Figure 1.
